# The Association of the 24 Hour Distribution of Time Spent in Physical Activity, Work, and Sleep with Emotional Exhaustion

**DOI:** 10.3390/ijerph15091927

**Published:** 2018-09-05

**Authors:** Janina Janurek, Sascha Abdel Hadi, Andreas Mojzisch, Jan Alexander Häusser

**Affiliations:** 1 Department of Psychology, Justus-Liebig-University Giessen, 35394 Giessen, Germany; Sascha.Abdel-Hadi@psychol.uni-giessen.de (S.A.H.); Jan.A.Haeusser@psychol.uni-giessen.de (J.A.H.); 2 Institute of Psychology, University of Hildesheim, 31141 Hildesheim, Germany; mojzisch@uni-hildesheim.de

**Keywords:** compositional analysis, multilevel analysis, sleep, physical activity, emotional exhaustion, burnout

## Abstract

Previous research identified time spent in physical activity, sleeping, and working as predictors of emotional exhaustion. However, this research did not take into account the interdependence of these time-use components. Since daily time is limited to 24 h, time spent in one specific activity (e.g., sleep) cannot be used for any other activity (e.g., physical activity). We conducted a one-week daily sampling study to assess the compositional effects of physical activity, sleep, and work on emotional exhaustion. Since the sample consisted of 104 undergraduate students, work was operationalized as study time. Participants wore accelerometers for one week continuously to assess sleep and physical activity. Also, they filled in questionnaires on study time and emotional exhaustion every morning. Multilevel and compositional data analyses were conducted. The multilevel analysis revealed significant between- (*p* = 0.012) and within-level (*p* < 0.001) associations of study time with emotional exhaustion. The compositional approach showed that time spent in physical activity was negatively related to emotional exhaustion (*p* = 0.007), whereas time spent studying was positively related to emotional exhaustion (*p* = 0.003), relative to the remaining two time-use components. In conclusion, our results show that emotional exhaustion is not only associated with work-related factors, but also with off-job physical activity.

## 1. Introduction

Burnout is defined by “a state of physical, emotional and mental exhaustion that results from long-term involvement in work situations that are emotionally demanding” [1] (p. 501). It comprises three key dimensions: emotional exhaustion, feelings of cynicism and detachment from the job, and feelings of ineffectiveness and missing personal accomplishment [2,3]. Emotional exhaustion represents the core element of burnout, referring to “feelings of being overextended and depleted of one’s emotional and physical resources” [4] (p. 399). Therefore, researchers often focus exclusively on emotional exhaustion when examining burnout (cf. [5]).

Burnout affects not only employees individually by constituting a severe risk factor for mental health issues [6] but also the company as a whole by increasing organizational costs. For example, in a 5-year prospective study, illness-related absence in individuals scoring high in burnout amounted to 13.9 days versus 6.0 days in individuals reporting low burnout scores [7]. Originally, burnout was considered to occur in individuals who work in the service sector [8]. However, later it became clear that burnout also exists outside this field [9].

Various predictors for the development of emotional exhaustion have been identified, with work-related predictors being the most prominent. Research on the Job–Demand–Control Model [10,11] identified cognitive (e.g., time pressure), physical (e.g., heavy lifting), and emotional (e.g., customer contact) job demands as influential for psychological well-being [5]. Besides qualitative differences in work characteristics, the mere amount of time spent at work (i.e., the working hours) turned out to be a strong predictor of burnout. For example, nurses working in 12 h shifts have been found to experience more emotional exhaustion than their 8 h shift colleagues [12]. Also, the number of working hours per week has been found to be positively associated with emotional exhaustion, both in a physician sample [13] and in a sample of nurses [14]. Furthermore, research revealed that the reduction of working hours can decrease emotional exhaustion [15,16].

Although the original development of the burnout construct implies a work-related etiology, research also turned to identifying off-job activities as predictors [17]. In this context, sleep is an interesting factor for at least two reasons. First, from the perspective of how much time is spent in a certain type of behavior, sleep is a highly prevalent activity. Second, sleep is essential for maintenance of physiological and psychological functioning and long-term health [18,19]. According to the National Sleep Foundation, for healthy adults without sleep-related diseases, the appropriate sleep duration is between 7 h and 9 h [20]. Söderström et al. [21] conducted a prospective study and identified too little sleep (<6 h per night) at baseline as a severe risk factor for the development of burnout during the two subsequent years. This finding has been replicated in other studies [22].

Besides time spent working and sleeping, another activity received an increasing amount of attention as a predictor of emotional exhaustion—time spent in physical activity. Research indicates that a minimum of 30 min of moderate physical activity (e.g., riding a bike) on at least five days a week can help to promote and maintain health [23]. In line with this general finding, participants who reported levels of moderate to vigorous physical activity (MVPA) that exceeded the recommended minimum MVPA reported lower levels of burnout, as compared to individuals who failed to match the recommendation [24]. Also, research indicates that engagement in physical activity may lower the risk of developing burnout two years later [25]. Regarding the underlying mechanisms of this relation, it was shown that physical activity is related to positive affect [26] and, in combination with sufficient sleep, the revitalization of personal resources [27] and can also improve sleep quality [28] which might again contribute to the prevention of emotional exhaustion and burnout.

As outlined above, numerous empirical studies have investigated the effects of the three types of behavior (i.e., time spent working, sleeping, and being physically active) on emotional exhaustion or burnout. Surprisingly, however, in previous research the examination of the predictors was conducted independently (i.e., the three predictors were examined in separate studies). To the best of our knowledge, so far, no study has simultaneously used the three types of behavior as predictors of emotional exhaustion or burnout.

The present research aims to address this research gap. As daily time is limited to 24 h, the amounts of time spent in the different activities are not independent of each other: time used for a specific activity (e.g., sleep or work) cannot be used for any other activity (e.g., physical activity). As a consequence, studies focusing on only one type of predictor (e.g., the number of working hours) without simultaneously considering the two other types (e.g., leisure time physical activity and sleep) are likely to yield erroneous conclusions [29,30,31]; see also [32] for a discussion of compositional data structure and a theoretical framework to analyze this type of data. For example, it is conceivable that not high job demands (i.e., the number of working hours) per se lead to emotional exhaustion but rather the fact that high job demands are typically associated with less physical activity and less sleep. In conclusion, not only can each predictor itself have an association with emotional exhaustion—as outlined above—but also the composition of the different types of behavior carried out during a given 24 h period.

University students are a specific population of young adults as their working hours consist mostly of attending lectures and studying outside of lectures. Nonetheless, this “student work” has very similar characteristics as compared to regular work, regarding, for example, hierarchical structures or deadlines [33]. It can, therefore, be hypothesized that long studying hours, just as long working hours, act as a predictor for burnout. Research has already shown that students’ workload has a positive effect on burnout. This relationship holds for both subjectively perceived [34] and actual workload [35]. No previous studies have examined study time, sleep, and physical activity as simultaneous predictors of emotional exhaustion in university students, while acknowledging the compositional properties of these time-use components. Using both compositional data analyses and multilevel analyses, the present study is the first to examine the three types of behavior (work, sleep, and physical activity) as simultaneous predictors of emotional exhaustion.

The current study aims at answering the following questions: Are the amounts of time spent in sleep, physical activity, and study related to emotional exhaustion? Are there any differences in the daily composition of sleep, physical activity, and study time between people with low and with high emotional exhaustion? Are the amounts of time spent in sleep, physical activity, and study associated with emotional exhaustion on a daily level?

## 2. Materials and Methods

### 2.1. Design and Participants

We conducted a one-week daily sampling study to assess the relationship between physical activity, study time, and psychological well-being in undergraduate students, using a convenience sample. Guided by recommendations on sample size for multilevel analysis, suggesting a minimum of 50 observation units on Level 2 [36], we set our sample size at *N* = 104 participants (89% female, mean age = 22.48 years, SD = 4.32). As an inclusion criterion, participants had to be at least second-year students to ensure that they had already fully adapted to a daily student routine. The student sample was heterogeneous with the majority of participants (62.5%) attending psychology courses. Around 59% of the participants reported not working in part-time jobs, whilst the remaining 41.3% had an average workload of 4.24 h per week in their part-time jobs. As a compensation for participation, participants received either €25 or course credit. Data analyses were conducted after data collection was completed and no participants were excluded from the analysis. All subjects gave their informed consent for inclusion before they participated in the study. The study was conducted in accordance with the Declaration of Helsinki, and was approved by the local Ethics Committee of the University of Hildesheim, Hildesheim, Germany.

### 2.2. Procedure

Participants were recruited via e-mail or phone and scheduled for an appointment in our laboratory. Participants filled in a baseline questionnaire containing questions on demographic data. For the following eight days, participants wore an accelerometer continuously (see below for a detailed description) and answered paper-and-pencil questionnaires every morning regarding the previous day and night. These questions asked about in-bed time in the evening, out-of-bed time in the morning, time spent studying (in courses and individually), and emotional exhaustion. After one week the accelerometers were retrieved and participants were thanked, debriefed, and compensated. The current study was part of a larger study including additional questionnaires which are irrelevant for the current research question, and will, therefore, not be outlined in detail. Constructs measured additionally in the baseline assessment were chronic stress (stress index, [37]), life and study contentment (life and study contentment scale, [38]), study demands and control (job content questionnaire—studies, [37]), and resilience (resilience scale, [39]), as well as questions on consumption behavior and sports. Constructs measured additionally on a daily level were subjective sleep quality (Pittsburgh Sleep Quality Index, [40]) and study demands and control (job content questionnaire—studies, [37]).

### 2.3. Assessment of Composition of the Day

For the analyses, we considered the amounts of time spent in the following components of the 24 h day: sleep, moderate to vigorous physical activity (MVPA), and study time. Sleep and MVPA were assessed using accelerometry (*ActiSleep+*; ActiGraph LLC, Pensacola, FL, USA). Accelerometers are small, lightweight devices designed to monitor spatial movements in three dimensions [41]. Participants wore the accelerometers continuously (day and night) for eight days on the wrist of their nondominant body side. We chose to attach the devices to the nondominant body side in order to avoid overestimation of movement behavior, since the dominant arm is in general used more often than the nondominant arm [42]. It should be mentioned that wrist-worn accelerometers are likely to entail the risk of overestimating movement behavior as the wrist is the most active site during wakefulness being involved in rather inactive types of behavior in contrast to, for example, the waist [41]. Nevertheless, we chose to attach the accelerometers to the wrist to ensure better compliance to wearing the devices continuously [43]. The Choi wear time validation [44] then served as an additional compliance check by identifying non-wear times. Following recommendations by Katapally and Muhajarine [45] that only data with a wear time ≥10 h a day should be considered, no data had to be excluded from analyses. The mean wear time per person per day amounted to 23.99 h in our study.

The marginal days that were not monitored completely (the first and the last day of data assessment) were excluded from analysis, leaving six full days of 24 h for each participant. The computation of times spent in sleep and MVPA was carried out using the software *ActiLife 6* [46]. Sleep periods were detected automatically by the software using established algorithms [47], and were adjusted with participants’ self-reports about their in-bed and out-of-bed times. Furthermore, for the computation of time spent in MVPA, every 60 s interval with ≥1952 counts (sample rate 30 Hz) was categorized as moderate to vigorous activity, using an algorithm developed by Freedson, Melanson, and Sirard [48]. Counts are derived by summing raw accelerometer data into epoch “chunks”, wherein the values of the counts vary depending on the frequency and intensity of the raw accelerometer data [49]. Study time was measured via self-report every morning retrospectively for the previous day. Participants answered the following two questions: “How much time did you spend at university attending lectures yesterday?” and “How much time did you spend studying apart from attending lecture yesterday (e.g., home exams, preparing for exams)?”. Participants indicated the respective times in hours. Both values were summed up to build the factor “study time”.

Times spent in each of the three types of behavior (sleep, MVPA, study) were transformed into minutes per day and were averaged over all six days of data assessment. These proportions of time spent in sleep, MVPA, and study were then expressed as percentages of 24 h so that their sum (+remaining, not-considered residual) equaled 100%.

### 2.4. Assessment of Emotional Exhaustion

To assess emotional exhaustion on a daily level, we constructed a German three-item short scale with items derived from two established scales for the assessment of burnout: The *Maslach Burnout Inventory* (MBI; [50]) and the *Oldenburg Burnout Inventory* (OLBI; [51]). Items were adapted to the day level—“Did you feel emotionally exhausted yesterday?”, “Did you feel worn out yesterday?”, and “Last night, did you have the feeling of not having done enough, although you worked hard?”—and answered on a five-point Likert scale. The internal consistency of the *emotional exhaustion* scale was Cronbach’s α = 0.85. To obtain information about what the composition of the day looked like for participants reporting high or low emotional exhaustion, we divided participants into two groups of different levels of emotional exhaustion using a median split. Since the scale for emotional exhaustion ranged from 1 to 5 with higher values representing higher emotional exhaustion, participants with values <2.22 (median) were categorized as “lowly emotionally exhausted” and participants with values >2.22 were categorized as “highly emotionally exhausted”.

### 2.5. Socio-Demographic Data and Covariates

Participants answered questions on socio-demographic variables (age, gender, children, field of study, semester, part-time job) during the first appointment in our laboratory directly before starting their one-week data assessment. In all following analyses, we included age and gender as covariates.

### 2.6. Data Analysis Strategy

Data analysis followed the compositional data approach suggested by Chastin et al. [29]. As an appropriate method of descriptive analysis of compositional data, the compositional mean was calculated. Furthermore, a variation matrix was used as a measure of dispersion. It shows the variability structure of the data by means of log-ratio variances (variances of the logs of all pairwise ratios between types of behavior), thereby accounting for the interdependent nature of compositional data [29].

The daily proportions of time spent in sleep, MVPA, and study, as well as the daily measurements of emotional exhaustion, were averaged per person over all six days of data assessment. For the examination of the associations between proportions of time spent in the three types of behavior and emotional exhaustion, linear regression models were then computed.

In order to take the interdependence of the three different types of behavior into account, a compositional analysis based on isometric log-ratio (ilr) transformation (adapted from [52], as suggested by [29]) was implemented. In this approach, the composition of the daily time spent in sleep, MVPA, and study, rather than the individual types of behavior, acts as the predictor variable [29]. All compositional analyses were conducted using the open source software *Physical Activity CoDa Regression Model* (PACRM) developed by McGregor et al. [53].

Our study provides day-level information over the course of one week with a nested data structure (days nested in persons). Therefore, we further conducted a stepwise two-level hierarchical analysis using raw data. As day-level data (Level 1) are nested within the person level (Level 2) in our study, this procedure accounts for the interdependent nature of the two levels [54]. In the multilevel analysis, regarding MVPA and study time, data from the same six days were used as in the compositional analysis, whereas sleep data were used from each previous night. To estimate variance components on both levels, we started with calculating an intercept-only model (null model) for emotional exhaustion. Model 1 includes the control variables age and gender (on Level 2), the Level 1 predictor variables (i.e., sleep duration, MVPA, and study time), and the averaged continuous Level 1 predictor variables to estimate the between-person relationships (Level 2). Finally, in a more exploratory manner, in Model 2, we additionally entered the interaction terms of the predictor variables of sleep duration, MVPA, and study time. We centered all day-level variables at the respective person mean and person-level variables at the grand mean (cf. [55]). All models are presented with random intercepts and fixed slopes. All multilevel analyses were conducted using *Stata/IC 15.1* [56].

All multivariate analyses were adjusted for age and gender. Furthermore, regression assumptions were examined a priori. Table 1 displays means, standard deviations, and correlations between the variables. Predictors’ means and standard deviations on Level 2 can be found in Table 2.

## 3. Results

### 3.1. Descriptive Statistics

Standard and compositional descriptive statistics of the proportions of time (percentage of 24 h) spent in the three types of behavior (sleep, MVPA, study) were calculated and are displayed in Table 2. The compositional means are smaller compared with standard arithmetic means, which indicates an overestimation by standard descriptive statistics in compositional data. For example, the mean relative amount of time spent studying is overestimated by the arithmetic mean as compared with the compositional mean by 1.63% of the day, that is, by approximately 23 min a day.

As a measure of dispersion for compositional data, a variation matrix was calculated (see Table 3), where the variability of the data is represented by means of pairwise log-ratio variances. If a value is close to zero, the times spent in the two types of behavior involved in the ratio are highly proportional [29]. The variance of log(sleep/MVPA) = 0.12 is closest to zero in comparison with the other pairs of types of behavior. Thus, the highest proportional relationship or interdependence in the data exists between sleep and MVPA.

**Figure 1:**
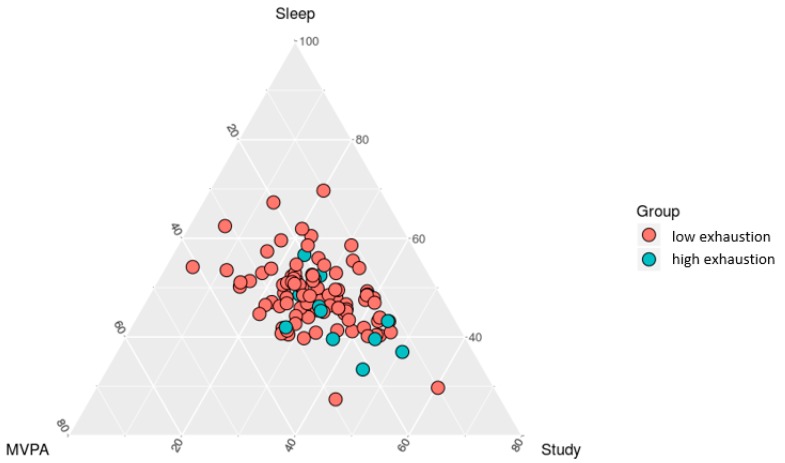
Ternary plot of the sample composition of time spent in sleep, MVPA, and study. Colored points represent groups of low (**red**) and high emotional exhaustion (**blue**).

Equivalent to scatterplots in standard descriptive statistics, ternary plots provide an overview of the distribution of compositional data [29]. Figure 1 displays a ternary plot of the distribution of the sample composition of time spent in sleep, MVPA, and study. Participants with low (red) and high (blue) emotional exhaustion are displayed in different colors.

### 3.2. Composition of the Day by Groups

**Figure 2:**
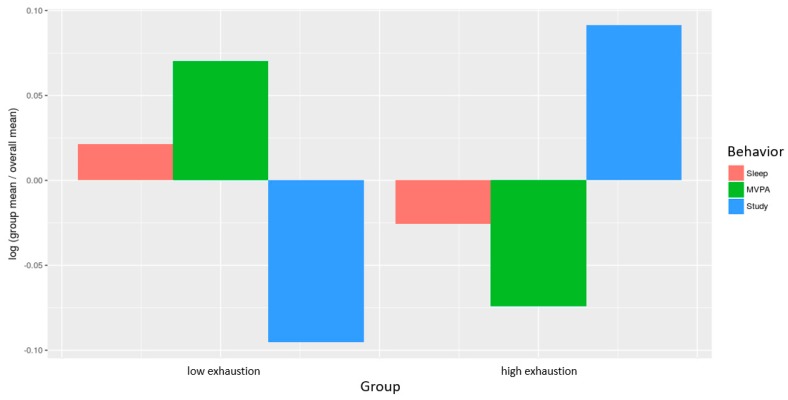
Composition of the day by groups of low and high emotional exhaustion (median split) as compositional analysis of the relative importance of the group mean time spent in sleep, MVPA, and study with respect to the overall mean time composition. The *y* axis displays the log-ratio value.

The composition of the day for the two different groups of lowly emotionally exhausted and highly emotionally exhausted participants is shown in Figure 2. For the compositional analysis of the relative distribution of times spent in the three types of behavior, for each group, the log-ratio between the group compositional mean and the overall compositional mean of the complete sample after centering the data was used [29]. Results show that for highly emotionally exhausted participants, time spent studying is around 9% higher (exp (0.09) = 1.094), whereas for lowly emotionally exhausted participants it is around 9% lower relative to the overall mean composition. For MVPA, an opposite effect occurs: for highly emotionally exhausted participants, time spent in moderate to vigorous physical activity is around 7% lower, whereas for lowly emotionally exhausted participants, it is around 7% higher relative to the overall mean composition.

### 3.3. Linear Regression Models: Compositional Analysis 

The model with age and gender as covariates and proportions of sleep, MVPA, and study time as predictor variables explains the variance for emotional exhaustion (R^2^ = 0.09). As a compositional method of analysis, the proportion of time spent in each type of behavior relative to the other two types of behavior was computed, and its impact on emotional exhaustion was tested. The proportion of time spent in MVPA relative to the other two types of behavior was significantly negatively related to emotional exhaustion (β = −0.29, *p* = 0.007), whereas the proportion of time spent studying was significantly positively related to emotional exhaustion (β = 0.45, *p* = 0.003). By contrast, there was no significant association between the proportion of time spent sleeping (relative to the other two types of behavior) and emotional exhaustion (β = −0.16, *p* = 0.141).

### 3.4. Linear Regression Models: Multilevel Analysis

Before conducting multilevel analyses, we calculated intraclass correlations to examine if emotional exhaustion varied within persons. Dividing the total variance into variance between and within persons showed that 52% of the total variance of emotional exhaustion can be attributed to within-person variations. Hence, in our study, a substantial part of the total variance lies within persons, emphasizing the volatility of emotional exhaustion and the benefit of an additional multilevel analysis (cf. [57]). Table 4 shows information on model fit (differences of −2 × log) as well as estimates for fixed and random parameters. Model 1, which contained the control variables age and gender as well as the predictor variables sleep duration, MVPA, and study time (on both Levels 1 and 2), showed significantly better model fit than the null model, which only contained the intercept, ∆−2 × log = 22.11; df = 8; *p* < 0.001. Study time was a positive predictor of emotional exhaustion on the day level (b = 0.001, *p* < 0.001) and on the person level (b = 0.002, *p* = 0.012). However, sleep duration and MVPA did not show significant associations with emotional exhaustion (sleep Level 1: b = −0.000, *p* = 0.240, Level 2: b = −0.000, *p* = 0.795; MVPA Level 1: b = −0.001, *p* = 0.173, Level 2: b = 0.001, *p* = 0.377). For exploratory analyses, Model 2 additionally included interaction terms for the predictors, but did not show significantly better model fit than Model 1, ∆−2 × log = 4.21; df = 6; *p* = 0.209. Study time still acted as a significant predictor of emotional exhaustion on Level 1, b = 0.001, *p* < 0.001, but not on Level 2, b = 0.001, *p* = 0.055. No other main effects were shown to be significant, and significant interaction effects were found neither between nor within persons (all *p* > 0.05).

## 4. Discussion

Research has identified time spent in physical activity, sleeping, and working as important predictors of emotional exhaustion and burnout [13,21,25]. The present study is the first to examine these three types of behavior as simultaneous predictors of emotional exhaustion. Since standard regression analyses are not able to take into account the compositional nature of such a data structure, a compositional approach based on isometric log-ratio (ilr) transformation was used [29,52].

Our results show that the amount of time spent physically active (relative to the amount of time spent sleeping or studying) was significantly negatively related to emotional exhaustion. This finding is perfectly in line with earlier theorizing and empirical findings, supporting the assumption that physical activity may prevent burnout [24,58]. Furthermore, the amount of time spent studying (relative to the amount of time spent sleeping or in physical activity) was significantly positively related to emotional exhaustion. Thus, our study supports the hypothesis that long working hours may increase the risk of developing burnout symptoms [12]. By contrast, the results do not show any significant relation between the amount of time spent sleeping and emotional exhaustion, although sleep has already been shown to have an effect on mental health in earlier research [21]. Potential reasons for this inconsistency will be discussed in Section 4.1.

Since our study also provides day-level information over the course of one week, we conducted an additional stepwise multilevel analysis. On a more general level, this approach also contributes to the literature on effects of job demands on emotional exhaustion, as studies conducted in this field typically adopt a between-person perspective (see [5] for an overview). The day-level approach used in our study accounts for intra-individual fluctuations in work characteristics and well-being. The results of the multilevel analyses revealed that study time is significantly related to emotional exhaustion on both the person level and the day level. Hence, individuals spending higher amounts of time studying are at higher risk of developing emotional exhaustion. However, also within persons, long study days increase the immediate experience of emotional exhaustion. By contrast, neither sleep nor physical activity showed significant effects on emotional exhaustion.

In summary, both compositional and multilevel analyses show that the amount of time spent studying (attending lectures as well as studying apart from lectures) acts as a significant predictor for emotional exhaustion. By contrast, regarding physical activity, results from compositional analyses and multilevel analyses diverge: physical activity was a significant predictor variable for emotional exhaustion in the compositional analysis, but not in the multilevel analysis. Albeit speculatively, a possible explanation could be that physical activity does not instantly affect emotional exhaustion. Instead, physical activity may pay off in the long run: by regularly engaging in physical activity at any time during the week, one may build and maintain a steady level of fitness and resilience, which, in turn, may prevent the detrimental effects of (job) stress on psychological strain, as shown by, for example, Schmidt et al. [59]. It is further conceivable that the correlative relationship between physical activity and emotional exhaustion shown in the compositional analysis results from a reverse causation: people suffering from mental strain may overall engage less in physical activity [60].

Previous research has already shown that workload in student samples, similar to regular work, has an impact on burnout [34,35]. Our study contributes to and extends these findings by showing that study time is positively associated with emotional exhaustion. In addition to studies identifying job characteristics as influential for psychological well-being [5], and especially the amount of time spent working [12], other studies suggest that off-job activities are also powerful predictors of burnout [17]. Our results support this multicausal perspective on burnout by showing that off-job physical activity is negatively related to emotional exhaustion. The current study thereby emphasizes the importance of a healthy work–life balance and engagement in off-job activities, especially physical activity, as a compensation for work.

### 4.1. Limitations

Some limitations of our study have to be pointed out. The first limitation concerns the time lags between the predictor variables. To recap, participants filled in the questionnaires every morning regarding their study time on the previous day as well as emotional exhaustion on the previous evening. Time spent sleeping and in physical activity were continuously assessed using accelerometry. Conducting multilevel analysis, we examined the effects of time spent sleeping the previous night as well as time spent in physical activity and study during the ongoing day on emotional exhaustion on the subsequent evening. Therefore, the time lags between the different predictor variables and the outcome variable are not equally long in duration. Although it has to be noted that in daily diary studies different time lags are common, this may still have influenced the impact of the predictors under study and contributed to the fact that our results are not completely in line with earlier research, showing no significant effects of sleep and physical activity on emotional exhaustion. For example, sleep might be more predictive for well-being in the morning (cf. [55]) as compared to well-being in the evening whereas study duration should be more predictive for well-being before going to bed.

Another limitation of our study is that rather low levels of emotional exhaustion were reported in our sample. For the illustration of the composition of the day by groups of lowly and highly emotionally exhausted participants, we therefore decided to use a median split of the emotional exhaustion values. A partition of the sample by the center of the scale would not have been reasonable since the “highly emotionally exhausted” group would have contained considerably fewer participants (*N* = 12) than the “lowly emotionally exhausted” group (*N* = 92). This circumstance may be another reason for the absent effects of sleep on emotional exhaustion in the compositional analysis as well as of sleep and physical activity in the multilevel analysis. While study time is closely linked to emotional exhaustion—both conceptually and regarding measurement with self-report questionnaires—sleep and MVPA are not. Sleep and MVPA might be of particular importance as resilience and recovery factors when emotional exhaustion is more pronounced. In contrast, differences in study time might already have effects on low to medium levels of burnout, as it more directly translates into (self-reported) exhaustion. Although university students are a specific population of young adults, the prevalence of sleep deprivation was not unusually high as compared to a working population [61]. Since the average sleep duration of the study sample was 6.68 h per night, it is rather unlikely that the absence of relationships is due to floor effects in levels of sleep.

Furthermore, it should be mentioned that wrist-worn accelerometers are likely to overestimate movement behavior (see [62] for a comparison of step counts between waist- and wrist-worn accelerometers) as the wrist is the most active body part during wakefulness, being also involved in rather inactive behaviors in contrast to the waist [41]. Nevertheless, we decided to attach the accelerometers to the wrist as we assumed higher acceptance of and compliance to wearing the devices continuously during a whole week [43]. For the analysis of sleep and physical activity, we used an algorithm, developed by Freedson and colleagues [48], that is suitable for the wrist as the wear site on the recommendation of the manufacturer (personal e-mail communication with ActiGraph [63] on 20 March 2018). Still, we cannot rule out that physical activity measured in our study is somewhat overestimated and noisy (which might also explain the lack of an effect in the multilevel analyses).

Finally, study time and MVPA are not necessarily mutually exclusive time-use components, as time spent in MVPA and time spent studying may overlap. However, since there were no sports students included in our sample (or other students with physical exercise classes), we assume that there should be only little overlap between these two factors in our study.

### 4.2. Implications for Future Research and Practice

Our study focused on the compositional effect of time spent in physical activity, sleep, and study on emotional exhaustion. Although compositional analysis makes it possible to examine time spent in different activities during a 24 h period, we did not examine every single behavior carried out that day. The accelerometers used are indeed capable of monitoring a whole period of 24 h, splitting the day into time spent sleeping, sedentary, and in light, moderate, and vigorous physical activity. In our study, we examined time spent studying which should have considerable overlaps with sedentary behavior. We, therefore, did not examine all possible classifications the accelerometry provides, but rather focused on self-reported study time, sleep, and moderate to vigorous physical activity. Nevertheless, future research could investigate the effects of 24 h movement behavior, including sleep, sedentary time, and time spent in light, moderate, and vigorous physical activity, on emotional exhaustion. Chastin and colleagues [29], among others [64,65], already examined the associations of 24 h movement behavior with physical health. To our knowledge, no studies have been conducted examining the effects of 24 h movement behavior on psychological well-being and mental health in healthy adults so far (see [66] for a study with adolescents). More generally, we like to emphasize that when examining movement behavior, it is important to account for the psychological quality of the specific behavior. For example, sedentary behavior is present while relaxing in an armchair, as well as while having a stressful job interview. Vigorous physical activity is present while jogging, as well as while running for the train (or away from the notorious sabretooth tiger). Hence, although identical from a mere physiological perspective, the specific reason for a physical activity might moderate its psychological effect on well-being and health. We strongly recommend future studies to account not only for the quantity and intensity of movement behavior but to also take reasons for movement and psychological quality into account. 

The sample of the current study consisted of undergraduate students who overall scored rather low on emotional exhaustion, as outlined above. A suggestion for future research is to examine study populations with a broader range of emotional exhaustion, which would allow us to investigate whether factors that are more indirectly (e.g., via recovery) linked to emotional exhaustion, such as sleep, are of better predictive value when it comes to the upper end of the exhaustion continuum. Furthermore, the gender distribution in our student sample was unbalanced (89% female). Due to the small case numbers of male participants, we were not able to conduct meaningful analyses of gender effects. However, we have no (theoretical) reason to assume considerable gender effects regarding the interplay between study time, physical activity, and emotional exhaustion. Nonetheless, future studies should ensure an equal distribution of female and male participants.

Our findings are also of practical relevance. Our results suggest that it may be beneficial for mental health to carefully determine the appropriate amount of time spent working. Research has shown that a restriction of working hours can decrease emotional exhaustion [15,16]. Our study highlights that the same applies to workload in students. Hence, when developing study schedules at universities, it should be taken into account that studying is structurally similar to work and care should be taken to keep the workload at a level that does not entail the risk of producing burnout. Furthermore, our results highlight the importance of time spent in physical activity for psychological well-being. It is, therefore, advisable to meet the public health recommendations for physical activity and engage in a minimum of 30 min of moderate physical activity on at least five days a week [23]. Universities and companies should be encouraged to provide possibilities to be physically active, for example, in the form of gym courses, running groups, or bike sharing programs to support students’ and employees’ health. On a societal level, policy-makers are challenged to develop work regulations that are suited to maintaining psychological well-being and that reduce work–life interferences.

## 5. Conclusions

In summary, our results show that considering the interdependent nature of different activities carried out during the day (i.e., sleeping, studying, and being physically active), time spent in physical activity is negatively related to emotional exhaustion. In addition, study time is positively associated with emotional exhaustion, both on a between-person level and on a day level within a person. In conclusion, these findings suggest that emotional exhaustion is not only associated with work-related factors, but also with off-job activities—more specifically, engagement in physical activity. As a consequence, the composition of one’s day is important for the prevention of emotional exhaustion and, therefore, for the maintenance of psychological well-being and mental health.

## Figures and Tables

**Table 1:** Means, standard deviations, and correlations between study variables.

		SD	1	2	3	4	5	6
**1. Sleep (Level 1, in minutes)**	M = 400.57	79.92	-	**−0.27**	−0.06	−0.03		
**2. MVPA (Level 1, in minutes)**	M = 184.58	75.12	**−0.31**	-	**−0.23**	**−0.13**		
**3. Study (Level 1, in minutes)**	M = 259.95	183.73	−0.09	**−0.30**	-	**0.26**		
**4. Emotional Exhaustion (Level 1)**	M = 2.25	0.92	−0.01	−0.17	**0.29**	-		
**5. Age**	M = 22.48	4.32	−0.07	0.18	−0.04	−0.05	-	
**6. Gender: Female**	% = 89		−0.13	−0.10	−0.06	−0.02	−0.05	-

Note: Correlations below the diagonal are person-level correlations (*N* = 104). Correlations above the diagonal are day-level correlations (*N* = 624). Correlations highlighted in bold are significant at *p* < 0.01. MVPA: Moderate to vigorous physical activity. M: mean; SD: standard deviation.

**Table 2:** Standard and compositional descriptive measures of the proportions of time spent in sleep, MVPA, and studying: arithmetic mean (standard deviation in parentheses) and compositional mean. Results expressed in percentage of 24 h.

	Sleep	MVPA	Study
Arithmetic Mean	27.91% (2.79)	12.82% (3.77)	18.05% (7.42)
Compositional Mean	27.77%	12.25%	16.42%

**Table 3:** Compositional variation matrix of time spent in sleep, MVPA, and study: pairwise log-ratio variances.

	Sleep	MVPA	Study
**Sleep**	0	0.12	0.24
**MVPA**	0.12	0	0.40
**Study**	0.24	0.40	0

**Table 4:** Multilevel estimates for models predicting day-level emotional exhaustion.

	Null Model		Model 1		Model 2	
Variable	b	SE	b	SE	b	SE
**Intercept**	2.254 **	0.068	2.408 **	0.434	2.331 **	0.425
**Age**			−0.005	0.015	−0.005	0.015
**Gender**			−0.046	0.218	−0.013	0.215
**Sleep (Level 1)**			−0.000	0.000	−0.001	0.000
**Sleep (Level 2)**			−0.000	0.002	−0.000	0.002
**MVPA (Level 1)**			−0.001	0.001	−0.001	0.001
**MVPA (Level 2)**			0.001	0.001	0.002	0.001
**Study (Level 1)**			0.001 **	0.000	0.001 **	0.000
**Study (Level 2)**			0.002 *	0.001	0.001	0.001
**Sleep (Level 1) × MVPA (Level 1)**					−0.000	0.019
**Sleep (Level 2) × MVPA (Level 2)**					0.000	0.000
**MVPA (Level 1) × Study (Level 1)**					−0.013	0.010
**MVPA (Level 2) × Study (Level 2)**					−0.004	0.000
**Study (Level 1) × Sleep (Level 1)**					−0.003	0.007
**Study (Level 2) × Sleep (Level 2)**					−0.000	0.000
**−2 × log(lh)**	−729.91		−707.80		−703.59	
**Difference of −2 × log(lh)**			22.11 **		4.21	
**df**			8		14	

Note: * *p* < 0.05. ** *p* < 0.001.

## References

[1] Schaufeli W.B., Greenglass E.R. (2001). Introduction to special issue on burnout and health. Psychol. Health.

[2] Maslach C., Jackson S.E. (1981). The measurement of experienced burnout. J. Organ. Behav..

[3] Maslach C., Leiter M.P. (2016). Understanding the burnout experience: Recent research and its implications for psychiatry. World Psychiatry.

[4] Maslach C., Schaufeli W.B., Leiter M.P. (2001). Job burnout. Annu. Rev. Psychol..

[5] Häusser J.A., Mojzisch A., Niesel M., Schulz-Hardt S. (2010). Ten years on: A review of recent research on the Job Demand–Control (-Support) model and psychological well-being. Work Stress.

[6] Maslach C. (2001). What have we learned about burnout and health?. Psychol. Health.

[7] Borritz M., Rugulies R., Bjorner J.B., Villadsen E., Mikkelsen O.A., Kristensen T.S. (2006). Burnout among employees in human service work: Design and baseline findings of the PUMA study. Scand. J. Public Health.

[8] Maslach C., Schaufeli W.B., Schaufeli W.B., Maslach C., Marek T. (1993). History and conceptual specificity of burnout. Professional Burnout: Recent Developments in Theory and Research.

[9] Maslach C., Leiter M.P. (1997). The Truth about Burnout.

[10] Karasek R.A. (1979). Job demands, job decision latitude, and mental strain: Implications for job redesign. Adm. Sci. Q..

[11] Karasek R.A., Theorell T. (1990). Healthy Work, Stress, Productivity, and the Construction of the Working Life.

[12] Iskera-Golec I., Folkard S., Marek T., Noworol C. (1996). Health, well-being and burnout of ICU nurses on 12- and 8-h shifts. Work Stress.

[13] Nishimura K., Nakamura F., Takegami M., Fukuhara S., Nakagawara J., Ogasawara K., Ono J., Shiokawa Y., Miyachi S., Nagata I. (2014). Cross-sectional survey of workload and burnout among Japanese physicians working in stroke care: The nationwide survey of acute stroke care capacity for proper designation of comprehensive stroke center in Japan (J-ASPECT) study. Circ. Cardiovasc. Qual. Outcomes.

[14] Park S., Lake E.T. (2005). Multilevel modeling of a clustered continuous outcome: Nurses’ work hours and burnout. Nurs. Res..

[15] Gopal R., Glasheen J.J., Miyoshi T.J., Prochazka A.V. (2005). Burnout and internal medicine resident work-hour restrictions. Arch. Intern. Med..

[16] Martini S., Arfken C.L., Balon R. (2006). Comparison of burnout among medical residents before and after the implementation of work hours limits. Acad. Psychiatry.

[17] Oerlemans W.G.M., Bakker A.B. (2014). Burnout and daily recovery: A day reconstruction study. J. Occup. Health Psychol..

[18] Åkerstedt T. (2006). Psychosocial stress and impaired sleep. Scand. J. Work Environ. Health.

[19] De Lange A.H., Kompier M.A.J., Taris T.W., Geurts S.A.E., Beckers D.G.J., Houtman I.L.D., Bongers P.M. (2009). A hard day’s night: A longitudinal study on the relationships among job demands and job control, sleep quality and fatigue. J. Sleep Res..

[20] Hirshkowitz M., Whiton K., Albert S.M., Alessi C., Bruni O., DonCarlos L., Hazen N., Herman J., Katz E.S., Kheirandish-Gozal L. (2015). National Sleep Foundation’s sleep time duration recommendations: Methodology and results summary. Sleep Health.

[21] Söderström M., Jeding K., Ekstedt M., Perski A., Åkerstedt T. (2012). Insufficient sleep predicts clinical burnout. J. Occup. Health Psychol..

[22] Wolf M.R., Rosenstock J.B. (2017). Inadequate Sleep and Exercise Associated with Burnout and Depression among Medical Students. Acad. Psychiatry.

[23] World Health Organization (2010). Global Recommendations on Physical Activity for Health.

[24] Gerber M., Lang C., Feldmeth A.K., Elliot C., Brand S., Holsboer-Trachsler E., Pühse U. (2015). Burnout and Mental Health in Swiss Vocational Students: The Moderating Role of Physical Activity. J. Res. Adolesc..

[25] Jonsdottir I.H., Rödjer L., Hadzibajramovic E., Börjesson M., Ahlborg G. (2010). A prospective study of leisure-time physical activity and mental health in Swedish health care workers and social insurance officers. Prev. Med..

[26] Feuerhahn N., Sonnentag S., Woll A. (2014). Exercise after work, psychological mediators, and affect: A day-level study. Eur. J. Work Organ. Psychol..

[27] Nägel I.J., Sonnentag S. (2013). Exercise and sleep predict personal resources in employees’ daily lives. Appl. Psychol..

[28] Yang P.-Y., Ho K.-H., Chen H.-C., Chien M.-Y. (2012). Exercise training improves sleep quality in middle-aged and older adults with sleep problems: A systematic review. J. Physiother..

[29] Chastin S.F.M., Palarea-Albaladejo J., Dontje M.L., Skelton D.A. (2015). Combined Effects of Time Spent in Physical Activity, Sedentary Behaviors and Sleep on Obesity and Cardio-Metabolic Health Markers: A Novel Compositional Data Analysis Approach. PLoS ONE.

[30] Dumuid D., Stanford T.E., Martin-Fernández J.-A., Pedišić Ž., Maher C.A., Lewis L.K., Hron K., Katzmarzyk P.T., Chaput J.P., Fogelholm M. (2017). Compositional data analysis for physical activity, sedentary time and sleep research. Stat. Methods Med. Res..

[31] Pedišić Ž. (2014). Measurement issues and poor adjustments for physical activity and sleep undermine sedentary behaviour research—The focus should shift to the balance between sleep, sedentary behaviour, standing and activity. Kinesiology.

[32] Pedišić Ž., Dumuid D., Olds T.S. (2017). Integrating sleep, sedentary behaviour, and physical activity research in the emerging field of time-use epidemiology: Definitions, concepts, statistical methods, theoretical framework, and future directions. Kinesiology.

[33] Cotton S.J., Dollard M.F., de Jonge J. (2002). Stress and student job design: Satisfaction, well-being, and performance in university students. Int. J. Stress Manag..

[34] Jacobs S.R., Dodd D. (2003). Student Burnout as a Function of Personality, Social Support, and Workload. J. Coll. Stud. Dev..

[35] Yang H.-J. (2004). Factors affecting student burnout and academic achievement in multiple enrollment programs in Taiwan’s technical–vocational colleges. Int. J. Educ. Dev..

[36] Nezlek J.B., Schröder-Abé M., Schütz A. (2006). Mehrebenenanalysen in der psychologischen Forschung. Psychol. Rundsch..

[37] Schmidt L., Obergfell J. (2011). Zwangsjacke Bachelor?! Stressempfinden und Gesundheit Studierender: Der Einfluss von Anforderungen und Entscheidungsfreiräumen bei Bachelor-und Diplomstudierenden nach Karaseks Demand-Control-Modell.

[38] Holm-Hadulla R.M., Hofmann F.-H. (2007). Lebens-und Studienzufriedenheitsskala.

[39] Schumacher J., Leppert K., Gunzelmann T., Strauß B., Brähler E. (2005). Die Resilienzskala–Ein Fragebogen zur Erfassung der psychischen Widerstandsfähigkeit als Personmerkmal. Z. Klin. Psychol. Psychiatr. Psychother..

[40] Buysse D.J., Reynolds C.F., Monk T.H., Berman S.R., Kupfer D.J. (1989). The Pittsburgh Sleep Quality Index: A new instrument for psychiatric practice and research. Psychiatry Res..

[41] Tryon W.W., Luiselli J.K., Reed D.D. (2011). Actigraphy: The ambulatory measurement of physical activity. Behavioral Sport Psychology—Evidence-Based Approaches to Performance Enhancement.

[42] Dieu O., Mikulovic J., Fardy P.S., Bui-Xuan G., Béghin L., Vanhelst J. (2017). Physical activity using wrist-worn accelerometers: Comparison of dominant and non-dominant wrist. Clin. Physiol. Funct. Imaging.

[43] Rosenberger M.E., Haskell W.L., Albinali F., Mota S., Nawyn J., Intille S. (2013). Estimating activity and sedentary behavior from an accelerometer on the hip or wrist. Med. Sci. Sports Exerc..

[44] Choi L., Liu Z., Matthews C.E., Buchowski M.S. (2011). Validation of Accelerometer Wear and Nonwear Time Classification Algorithm. Med. Sci. Sports Exerc..

[45] Katapally T.R., Muhajarine N. (2014). Towards Uniform Accelerometry Analysis: A Standardization Methodology to Minimize Measurement Bias Due to Systematic Accelerometer Wear-Time Variation. J. Sports Sci. Med..

[46] ActiGraph L.L.C. (2012). ActiLife 6.

[47] Sadeh A., Sharkey M., Carskadon M.A. (1994). Activity-Based Sleep-Wake Identification: An Empirical Test of Methodological Issues. Sleep.

[48] Freedson P.S., Melanson E., Sirard J. (1998). Calibration of the Computer Science and Applications, Inc. accelerometer. Med. Sci. Sports Exerc..

[49] What Are Counts?. https://actigraph.desk.com/customer/en/portal/articles/2515580-what-are-counts-.

[50] Maslach C., Jackson S.E., Leiter M.P., Schaufeli W.B., Schwab R.L. (1986). Maslach Burnout Inventory.

[51] Demerouti E., Bakker A.B., Vardakou I., Kantas A. (2003). The convergent validity of two burnout instruments: A multitrait-multimethod analysis. Eur. J. Psychol. Assess..

[52] Hron K., Filzmoser P., Thompson K. (2012). Linear regression with compositional explanatory variables. J. Appl. Stat..

[53] McGregor D., Chastin S.F.M., Dall P., Palarea-Albaladejo J. (2018). Physical Activity CoDa Regression Model (PACRM).

[54] Hox J.J. (2002). Multilevel Analysis: Techniques and Applications.

[55] Sonnentag S., Binnewies C., Mojza E.J. (2008). “Did you have a nice evening?” A day-level study on recovery experiences, sleep, and affect. J. Appl. Psychol..

[56] StataCorp (2017). Stata Statistical Software: Release 15.

[57] Bakker A.B., Demerouti E., Sanz-Vergel A.I. (2014). Burnout and work engagement: The JD-R approach. Annu. Rev. Organ. Psychol. Organ. Behav..

[58] Häusser J.A., Mojzisch A. (2017). The physical activity-mediated Demand–Control (pamDC) model: Linking work characteristics, leisure time physical activity, and well-being. Work Stress.

[59] Schmidt K.-H., Beck R., Rivkin W., Diestel S. (2016). Self-control demands at work and psychological strain: The moderating role of physical fitness. Int. J. Stress Manag..

[60] Roshanaei-Moghaddam B., Katon W.J., Russo J. (2009). The longitudinal effects of depression on physical activity. Gen. Hosp. Psychiatry.

[61] Schoenborn C.A., Adams P.F. (2010). Health Behaviors of Adults: United States, 2005–2007.

[62] Tudor-Locke C., Barreira T.V., Schuna J.M. (2015). Comparison of step outputs for waist and wrist accelerometer attachment sites. Med. Sci. Sports Exerc..

[63] Willis A., Janurek J. (2018). Personal e-mail communication.

[64] Dumuid D., Pedišić Ž., Stanford T.E., Martín-Fernández J.-A., Hron K., Maher C.A., Lewis L.K., Olds T.S. (2017). The compositional isotemporal substitution model: A method for estimating changes in a health outcome for reallocation of time between sleep, physical activity and sedentary behaviour. Stat. Methods Med. Res..

[65] Hunt T., Williams M.T., Olds T.S., Dumuid D. (2018). Patterns of Time Use across the Chronic Obstructive Pulmonary Disease Severity Spectrum. Int. J. Environ. Res. Public Health.

[66] Wong M., Olds T.S., Gold L., Lycett K., Dumuid D., Muller J., Mensah F.K., Burgner D., Carlin J.B., Edwards B. (2017). Time-use patterns and health-related quality of life in adolescents. Pediatrics.

